# Supplementary material for: Predicting visual function by interpreting a neuronal wiring diagram
Source: Nature. 2024 Oct 2;634(8032):113–23. doi: 10.1038/s41586-024-07953-5 (PMC11446822; doi:10.1038/s41586-024-07953-5)
Supplement: Supplementary file 6 — CRF and ERF predictions for individual TmY4 and TmY9 cells. Analogous to Supplementary Data 3, but for TmY target types. Shown are the top four monosynaptic pathways, the strongest pathway passing through each of the top ten intermediary types (ranking from Extended Data Fig. 7), and the trisynaptic pathway Tm1–TmY–Dm3–TmY (see the section entitled Prediction of spatial normalization). [file 41586_2024_7953_MOESM6_ESM.zip › DataS4/TmY9q/720575940640202448.pdf]

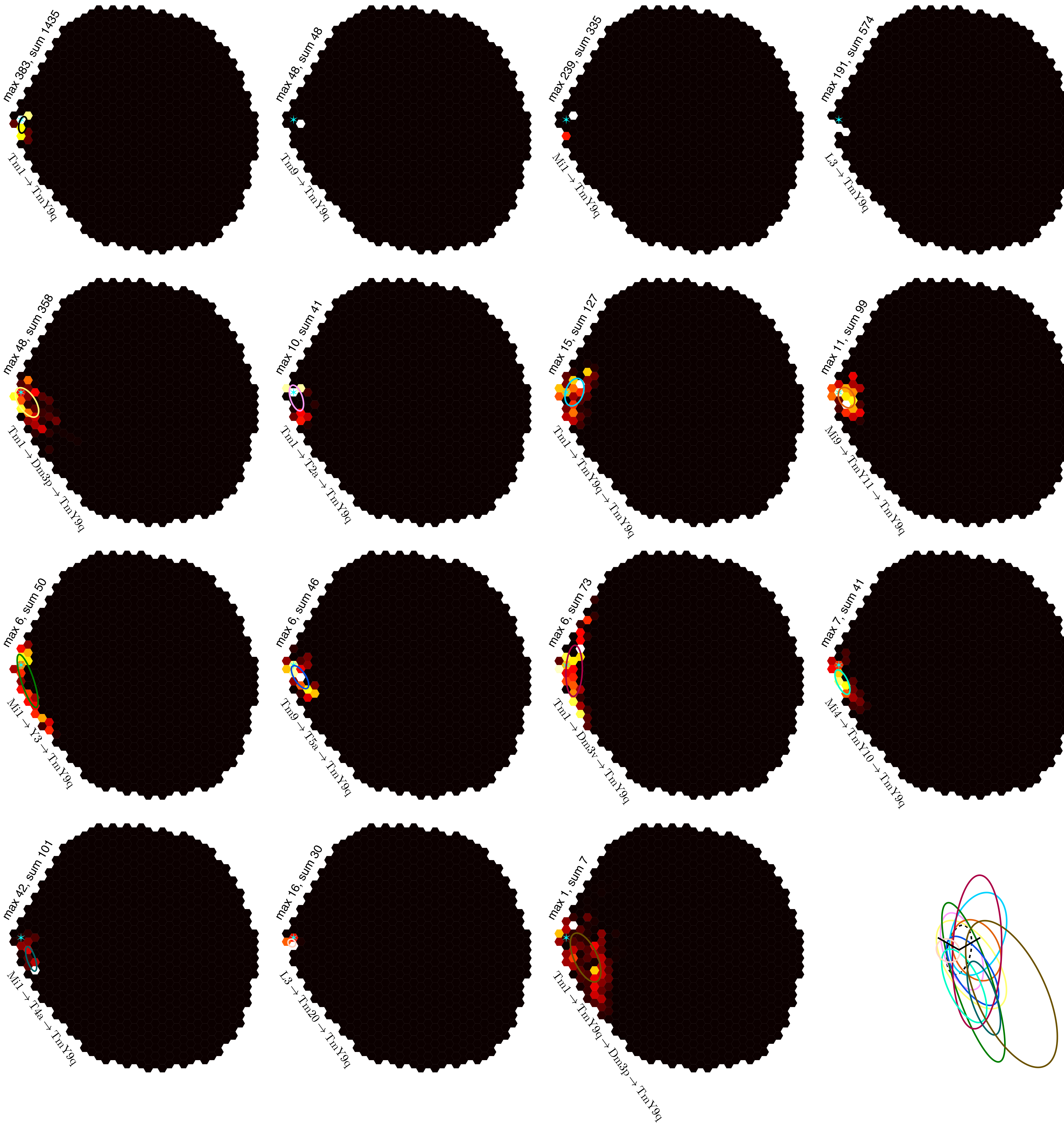

max 383, sum 1435
Tm1 → TmY9q
max 48, sum 48
Tm9 → TmY9q
max 239, sum 335
Mi1 → TmY9q
max 191, sum 574
L3 → TmY9q
max 48, sum 358
Tm1 → Dm3p → TmY9q
max 10, sum 41
Tm1 → T2a → TmY9q
max 15, sum 127
Tm1 → TmY9q → TmY9q
max 11, sum 99
Mi9 → TmY11 → TmY9q
max 6, sum 50
Mi1 → Y3 → TmY9q
max 6, sum 46
Tm9 → T5a → TmY9q
max 6, sum 73
Tm1 → Dm3v → TmY9q
max 7, sum 41
Mi4 → TmY10 → TmY9q
max 42, sum 101
Mi1 → T4a → TmY9q
max 16, sum 30
L3 → Tm20 → TmY9q
max 1, sum 7
Tm1 → TmY9q → Dm3p → TmY9q
